# Does Combination Therapy with Desmopressin and Tolterodine Improve the Treatment Outcomes of Patients with Monosymptomatic Nocturnal Enuresis? A Randomized Clinical Controlled Trial

**DOI:** 10.1155/2013/413146

**Published:** 2013-03-25

**Authors:** Fahimeh Kazemi Rashed, Davoud Nourizade, Sakineh Hajebrahimi, Kamaleddin Hasanzade, Abdolreza Otoofat

**Affiliations:** ^1^Urology Department, Imam Reza Hospital, Tabriz University of Medical Sciences, Azadi Avenue, Golgasht Street, Tabriz 5165665931, Iran; ^2^Urology Department, Sina Hospital, Tabriz University of Medical Sciences, Azadi Avenue, Tabriz 5163639888, Iran

## Abstract

Several therapeutic options have been described for children with nocturnal enuresis, but still their efficacy and outcomes are controversial. This study compares the combined Desmopressin and Tolterodine efficacy versus Desmopressin alone efficacy in the treatment of nocturnal enuresis. One hundred children 5–16 years old with nocturnal enuresis were enrolled in a randomized trial study and were assigned to two equal groups. In a double-blind manner, we used 2 mg of Tolterodine tablet plus 20 **μ**g of nasal Desmopressin in group A and 20 **μ**g of nasal Desmopressin plus placebo in group B. The two groups were matched for age and sex (*P* = 0.547, *P* = 0.414). The mean number of the wet nights was reduced in both groups (*P* < 0.001, *P* < 0.001). Upon ICCS scoring in the Tolterodine + Desmopressin group, 27 (54%) had full response, 17 (34%) had partial response, and 5 (10%) had an unsuccessful outcome. In the Desmopressin + placebo group, 17 (34%) had full response, 23 (46%) had partial response, and 10 (20%) had an unsuccessful outcome. The response in the Tolterodine + Desmopressin group was significantly higher (*P* = 0.049). Regarding the results, combined Tolterodine plus Desmopressin is slightly more effective than monotherapy.

## 1. Introduction

Primary nocturnal enuresis is intermittent nocturnal incontinence in children aged more than 5 years in the absence of structural and nervous system abnormalities. The overall prevalence of enuresis at the age of 5 years is about 15–20% and 1-2% in the age of 15 years [1]. 

Treatment may consist of certain medications, behavioral therapy, conditional therapy, or most often a combination of these approaches. Behavioral modification is recognized as the first step of management in enuresis. Although many approached have considered behavioral modification to enuresis, by far alarm units have counted as the most effective method [2]. It is known a successful outcome depends to appropriate compliance of the child and her or his parents. For this reason the most common cause of failure is their poor cooperation. Medication used for the treatment of enuresis includes Desmopressin, anticholinergic agents, and tricyclic antidepressants. Initial treatment with Desmopressin has been proven in various studies. Desmopressin improves enuresis in 65–80% patients, but refractory rate is 20–25% to Desmopressin [3]. Regarding the high prevalence of enuresis (15–20%) and the unsuccessful response to Desmopressin alone (25–20%) and critical side effects of tricyclic antidepressants, the need to identify a new strategy with minimal side effects and maximum effectiveness is felt. It is now generally accepted that the nocturnal urine output in many enuretic children is in excess of the bladder functional capacity during sleep at night or bladder instability [4]. Nocturnal polyuria can be either absolute which is usually associated with a dearrangement of the circadian rhythm of antidiuretic hormone (ADH) secretion or relative, mainly due to a reduced functional bladder capacity during sleep at night [5].

It is clear that there is no exact method to cure the enuresis, and Desmopressin alone cannot be completely effective in patients with enuresis; we designed a study to evaluate the effect of Desmopressin plus Tolterodine versus Desmopressin alone in the treatment of primary enuresis. Anticholinergic agents increase functional bladder capacity, and Desmopressin decreases polyuria. This combined treatment may have additive effect in the treatment of nocturnal enuresis. There are several reports of the combination therapy (anticholinergic + Desmopressin), but most of them considered small number of children, and no high quality randomized controlled trial is reported [6]. This RCT is designed to evaluate this combination therapy with larger group of patients in a controlled trial manner. 

## 2. Materials and Methods

The study was approved by the Ethical Committee of Tabriz University of Medical Sciences. Children aged from 5 to 16 years old with primary nocturnal enuresis who referred to Urology clinic of Tabriz University of Medical Sciences, Tabriz, Iran, were enrolled in the study from March to December 2011. Regarding definition of ICCS, patients with spine abnormality, genitourinary abnormality, history of lower urinary tract symptom, daytime incontinence, encopresis, constipation, urinary tract infection, neurogenic bladder, allergy toward anticholinergic agent, and epilepsy were excluded from the study. After history taking and physical examination, all patients were studied by urine analysis, urine culture, and urinary tract ultrasound. If the results were abnormal, they were excluded. In a randomized, double-blind controlled trial, 100 patients with primary enuresis were randomly divided into groups A and B. For random allocation, two types of envelopes were prepared. Envelopes A and B considered Tolterodine and Desmopressin and placebo with Desmopressin respectively. Patients and outcome analyzer were blinded during research. Patients received randomly a medication with special envelope. The researcher and patients were not informed about taking what kind of envelope. Patients were asked to take 1 tablet each night, with their nasal spray Desmopressin for 4 weeks. In the end of the treatment period, all children were controlled for medication taken. Upon special envelopes of medication, the patients were divided into two groups: A and B. Group A patients had been taking Tolterodine 2 mg tablets and nasal Desmopressin with a dose of 20 *μ*g, and group B patients had been taking placebo and nasal Desmopressin with a dose of 20 *μ*g. To compare the response to medication (short outcome or during the receiving medication), we used the definition of the Standardization Committee of the International Children continence Society as follows.

1. A full response, no wet nights.
2. A complete response to treatment (90% reduction in the number of enuresis episodes per week).
3. A partial response to treatment (from 50 to 89% reduction in the number of enuresis episodes per week).
4. Failure to respond to treatment (less than 50% reduction in the number of enuresis episodes per week).

During the study, one patient from the group of Desmopressin + Tolterodine was excluded from the study because of poor adherence in taking the medicine.

*T*-test was used to evaluate the quantitative data of the samples, and Mann-Whitney *U* test was used for qualitative data. Statistical analysis was down according to SPSS version 17.

## 3. Results

The demographic findings of patients are shown in Table 1. It shows that the two groups were matched for age and gender.

The mean wet nights for patients in the two groups are shown in Table 2.

**Figure 1:**
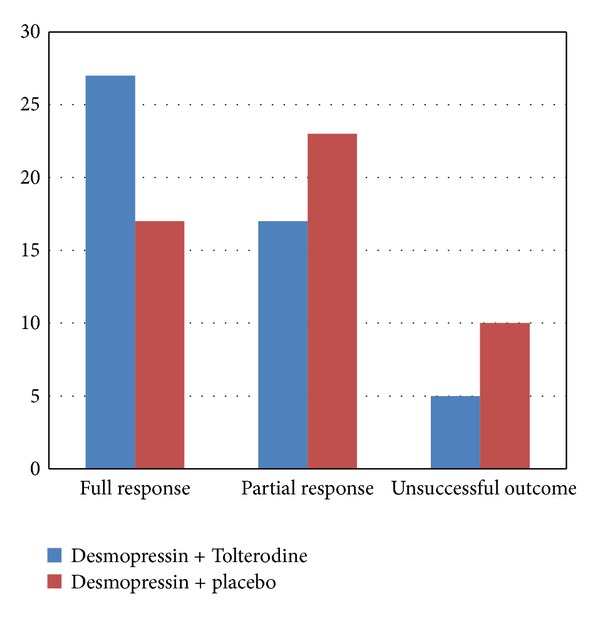
Response to the treatment upon CICCS scoring.

Based upon the values of the Committee of the International Children's Continence Society in the group treated with Desmopressin + Tolterodine, 27 patients (54%) had full response, 17 patients (34%) had partial response, and 5 patients (10%) had an unsuccessful response, and in the group treated with Desmopressin + placebo, 17 patients (34%) had full response, 23 patients (46%) had partial response, and 10 patients (20%) had an unsuccessful response. Comparing these two groups together shows that the full response rate in the group treated with Desmopressin + Tolterodine is significantly higher in comparison to the group which was treated with Desmopressin + placebo (*P* = 0.049). In the group treated with Desmopressin plus Tolterodine, in 27 patients (54%), the number of bedwetting has been reduced to zero, in 21 patients (42%), these episodes have been decreased, and in one patient (2%), no change has been observed. These values in Desmopressin + placebo group were as follows: in 17 cases (34%), the number of bedwetting has been reduced to zero, in 31 cases (62%), the episodes have been decreased, and in 2 patients (4%), no change has been observed (Figure 1). The difference between the two groups was statistically significant (*P* = 0.041). 

## 4. Discussion

Enuresis is one of the most prevalent disorders in children. Upon studies, there is a different prevalence of this disorder which differs from 15 to 20% in children higher than 5 years [1]. There are 2 subtypes of enuresis, the primary enuresis and the secondary one [2]. The primary enuresis is a type in which the patient had a history of enuresis from the birth time, and in secondary type, the patient was dry at least for the last 6 months [2]. Primary nocturnal enuresis in many cases is self-limited. When the patients are distressed by the night bedwetting and the wetting is associated with a loss of self-esteem, intervention is justified [7]. Enuresis can be also categorized as monosymptomatic or nonmonosymptomatic. Monosymptomatic enuresis occurs in the absence of any daytime voiding symptoms, such as frequency, urgency, or daytime incontinence. Nonmonosymptomatic enuresis is associated with daytime symptoms [2]. Nocturnal enuresis has no known etiology, and several factors are likely to contribute to its pathogenesis. A number of contributory factors including genetic ones, nocturnal polyuria, decreased functional bladder capacity or abnormal bladder function, sleep disorders, and psychological ones have been speculated [8]. There are some studies using urodynamic evaluation which demonstrate a high incidence of bladder instability—particularly in those who do not respond to Desmopressin [4]. Detrusor over activity at night is found with nocturnal cystometry in 35% of children with enuresis. Some studies have augmented that the reduction in nocturnal functional bladder capacity is a common factor in the pathogenesis of refractory nocturnal enuresis [9, 10]. These findings suggest that the combination therapy (Desmopressin + anticholinergic) may improve treatment of nocturnal enuresis. 

Cendron and Klauber examined the effect of Hyoscine and Desmopressin combination therapy on enuresis and found that in 78% of patients, this combination will improve the disease [6]. But with having more selective anticholinergic agents for lower urinary tract, Hyoscine has not been used in pediatric urology [11]. In 2001, Nevéus studied combined effects of Oxybutynin and Desmopressin on enuresis and found that this combination in 50% of cases will improve the disease [12]. In an experience by Triantafyllidis and coworkers for the managing of nocturnal enuresis in Greek children, they showed that the use of desmopressin, and anticholinergics (Oxybutynin or Tolterodine) in specific subgroups is effective and safe for the management of nocturnal enuresis in children [13]. Austin and his colleagues designed a randomized, double-blind, placebo-controlled study for the evaluation and comparison of the Desmopressin alone and Desmopressin plus Tolterodine effect on enuresis. They studied 41 patients with enuresis refractory to Desmopressin, and they concluded that the mean dry nights in Desmopressin + Tolterodine group was significantly lower than Desmopressin group alone. In their study, only patients with refractory enuresis to Desmopressin were enrolled [11], but in our research, each patient with primary monosymptomatic nocturnal enuresis was enrolled. Radvanska et al. examined the effect of combination therapy (Desmopressin + Oxybutynin) on enuresis and found a 43% reduction in the mean number of wet nights per week after the combination therapy [14].

In our study, the frequency of enuresis before and after treatment in Desmopressin + Tolterodine group shows significant difference, meaning that the number of wet nights after treatment with Desmopressin plus Tolterodine declined dramatically (*P* < 0.001). The results also show a significant reduce in wet nights after the treatment with Desmopressin + placebo (*P* < 0.001).

Based on the Committee of the International Children's Continence Society in the group treated with Desmopressin + Tolterodine, 27 patients (54%) had full response, 17 patients (34%) had partial response and 5 patients (10%) has an unsuccessful response. In the group treated with Desmopressin + placebo, 17 patients (34%) had full response, 23 patients (46%) had partial response, and 10 patients (20%) had an unsuccessful outcome. Comparing these two groups together shows that the full response rate in the group treated with Desmopressin + Tolterodine is significantly higher in comparison to the group which was treated with Desmopressin + placebo (*P* = 0.049).

These findings are compatible with the results of Austin and colleagues in which they reported a reduction to zero in bedwetting in 66% of cases treated with Desmopressin + Tolterodine [11]; this rate was 54% in our study.

Based on the results observed in this study, Desmopressin combined with placebo treatment had a success rate of 34% in full treatment of enuresis, while the combination of Desmopressin + Tolterodine had a 54% full treatment rate. In Desmopressin + placebo group, full and partial responses were 80% and in Desmopressin + Tolterodine group were 88%. In the other word number needed to treat for full response in combination therapy was 5[1/(54%–35%)]. It means that if we treat 5 children with this regime one is going to be cured just because of this kind of treatment, and we conclude that this method of combination can be reasonable. Other studies with bigger sample size and long-term followup are recommended. 

## 5. Conclusion

The results show that Tolterodine + Desmopressin work better in full treatment of enuresis in comparison with Desmopressin alone. With regard to these findings, we can use this combination (Desmopressin + Tolterodine) for children suffering from enuresis with high efficacy and minimal side effects. 

## Figures and Tables

**Table 1:** Age and gender of patients.

Variable	Group		*P* value
	Desmopressin + Tolterodine	Desmopressin + placebo
Age (mean)	9.26 ± 1.85	9.50 ± 1.68	0.547
Gender	50% male, 48% female	42% male, 58% female	0.414

**Table 2:** The mean wet nights before and after treatment in two groups.

Group	Mean wet nights before treatment per week (mean)	Mean wet nights after treatment per week (mean)	*P* value
Desmopressin + Tolterodine	3.89 ± 1.19	0.75 ± 1.05	<0.001
Desmopressin + placebo	3.94 ± 0.97	1.36 ± 1.43	<0.001

## References

[1] Foxman B, Valdez RB, Brook RH (1986). Childhood enuresis: Prevalence, perceived impact, and prescribed treatments. *Pediatrics*.

[2] Nevéus T, von Gontard A, Hoebeke P (2006). The standardization of terminology of lower urinary tract function in children and adolescents: report from the standardisation committee of the international children’s continence society. *Journal of Urology*.

[3] Terho P (1991). Desmopressin in nocturnal enuresis. *Journal of Urology*.

[4] Yeung CK, Chiu HN, Sit FKY (1999). Bladder dysfunction in children with refractory monosymptomatic primary nocturnal enuresis. *Journal of Urology*.

[5] Vande Walle J, Vande Walle C, Van Sintjan P (2007). Nocturnal polyuria is related to 24-hour diuresis and osmotic excretion in an enuresis population referred to a tertiary center. *Journal of Urology*.

[6] Cendron M, Klauber G (1998). Combination therapy in the treatment of persistent nocturnal enuresis. *British Journal of Urology*.

[7] Butler RJ, Redfern EJ, Holland P (1994). Children’s notions about enuresis and the implications for treatment. *Scandinavian Journal of Urology and Nephrology*.

[8] Nørgaard J, Djurhuus J (1993). The pathophysiology of enuresis in children and young adults. *Clinical Pediatrics*.

[9] Yeung CK, Sit FKY, To LKC (2002). Reduction in nocturnal functional bladder capacity is a common factor in the pathogenesis of refractory nocturnal enuresis. *British Journal of Urology international*.

[10] Medel R, Ruarte AC, Castera R, Podesta ML (1998). Primary enuresis: a urodynamic evaluation. *British Journal of Urology*.

[11] Austin PF, Ferguson G, Yan Y, Campigotto MJ, Royer ME, Coplen DE (2008). Combination therapy with desmopressin and an anticholinergic medication for nonresponders to desmopressin for monosymptomatic nocturnal Enuresis: a randomized, double-blind, placebo-controlled trial. *Pediatrics*.

[12] Nevéus T (2001). Oxybutynin, desmopressin and enuresis. *Journal of Urology*.

[13] Triantafyllidis A, Charalambous S, Papatsoris AG (2005). Management of nocturnal enuresis in Greek children. *Pediatric Nephrology*.

[14] Radvanska E, Kovács L, Rittig S (2006). The role of bladder capacity in antidiuretic and anticholinergic treatment for nocturnal Enuresis. *Journal of Urology*.

